# Sleep, major depressive disorder, and Alzheimer disease: A Mendelian randomization study

**DOI:** 10.1212/WNL.0000000000010463

**Published:** 2020-10-06

**Authors:** Jian Huang, Verena Zuber, Paul M. Matthews, Paul Elliott, Joanna Tzoulaki, Abbas Dehghan

**Affiliations:** From the MRC Centre for Environment and Health (J.H., V.Z., P.E., J.T., A.D.), Department of Epidemiology and Biostatistics, School of Public Health, St. Mary's Campus, Imperial College London, Norfolk Place; UK Dementia Research Institute at Imperial College London (J.H., P.M.M., J.T., A.D.); Imperial College NIHR Biomedical Research Centre (J.H., P.E.); Department of Brain Sciences (P.M.M., P.E.), Faculty of Medicine, Imperial College London; Health Data Research UK-London; and Department of Hygiene and Epidemiology (P.E., J.T.), University of Ioannina Medical School, Greece.

## Abstract

**Objective:**

To explore the causal relationships between sleep, major depressive disorder (MDD), and Alzheimer disease (AD).

**Methods:**

We conducted bidirectional 2-sample Mendelian randomization analyses. Genetic associations were obtained from the largest genome-wide association studies currently available in UK Biobank (n = 446,118), Psychiatric Genomics Consortium (n = 18,759), and International Genomics of Alzheimer's Project (n = 63,926). We used the inverse variance–weighted Mendelian randomization method to estimate causal effects and weighted median and Mendelian randomization–Egger for sensitivity analyses to test for pleiotropic effects.

**Results:**

We found that higher risk of AD was significantly associated with being a “morning person” (odds ratio [OR] 1.01, *p* = 0.001), shorter sleep duration (self-reported: β = −0.006, *p* = 1.9 × 10^−4^; accelerometer based: β = −0.015, *p* = 6.9 × 10^−5^), less likely to report long sleep (β = −0.003, *p* = 7.3 × 10^−7^), earlier timing of the least active 5 hours (β = −0.024, *p* = 1.7 × 10^−13^), and a smaller number of sleep episodes (β = −0.025, *p* = 5.7 × 10^−14^) after adjustment for multiple comparisons. We also found that higher risk of AD was associated with lower risk of insomnia (OR 0.99, *p* = 7 × 10^−13^). However, we did not find evidence that these abnormal sleep patterns were causally related to AD or for a significant causal relationship between MDD and risk of AD.

**Conclusion:**

We found that AD may causally influence sleep patterns. However, we did not find evidence supporting a causal role of disturbed sleep patterns for AD or evidence for a causal relationship between MDD and AD.

Aging populations across the world have led to an increasing prevalence of both Alzheimer disease (AD) and depression.^1–3^ The comorbidity of depressive disorders and late-life neurodegenerative diseases, including AD, has been widely reported.^4–6^ However, it is not known if any causal relationship exists between them or, alternatively, whether their co-occurrence is due to confounding or common risk factors such as aging. A genome-wide association study (GWAS) did not find evidence for a shared genetic architecture between depression and AD,^7^ supporting roles for common nongenetic risk factors. Sleep habits are important aspects of lifestyle, and abnormal sleep patterns are among the clinical signs and symptoms of both depression and AD. Sleep deprivation affects brain function and is associated with cognitive decline, anxiety, and depression.^8^ Clinical associations have been reported between major depressive disorder (MDD) and AD and are supported indirectly by their common effects on hippocampal atrophy and the involvement of molecular pathways related to oxidative stress in the progression of both diseases.^9^ However, potential causal relationships between sleep habits and MDD or AD have not been explored directly at a population level to the best of our knowledge.

We hypothesized that sleep causally affects MDD and AD but that there is no causal relationship between MDD and AD. To test these hypotheses, we investigated causal relationships in the triad of sleep, MDD, and AD using Mendelian randomization (MR) analyses. The MR approach overcomes unmeasured confounding and reverse causation in observational studies.^10^ Understanding these causal relationships will elucidate the mechanisms responsible for development of these diseases, better describe potential interactions between depression and AD, and further test the hypothesis that interventions to improve sleep patterns could modify disease risks.

## Methods

### Standard protocol approvals, registrations, and patient consents

This study used summary data published by multiple GWAS; patient consents were obtained by corresponding studies. This study is reported following the Strengthening the Reporting of Observational Studies in Epidemiology reporting guideline.

### Study design

We conducted a bidirectional 2-sample MR study to investigate causal relationships between 6 sleep-related phenotypes, MDD, and AD.

### Data sources

#### Sleep-related phenotypes

GWAS of sleep-related phenotypes were based on data from the UK Biobank, a population-based prospective study.^11^ We investigated sleep using a number of sleep-related phenotypes. The phenotypes either were self-reported (chronotype, insomnia, sleep duration) or were estimated with the accelerometer (the number of sleep episodes, sleep duration, least active 5 hours [L5] timing, and sleep efficiency). We chose instrumental variables and extracted summary statistics for the association of genetic variants with sleep-related phenotypes using a number of recent GWAS as listed in supplementary table e-1, available from Dryad, doi.org/10.5061/dryad.ffbg79cqt.

#### Chronotype

Chronotype is considered the proclivity for earlier or later timing of sleep.^12^ An individual who prefers going to bed and waking earlier is considered a “morning person,” while a person who prefer going to bed and waking later is considered an “evening person.”^12^ We obtained the genetic associations of self-reported chronotype from a GWAS among 403,195 individuals of European ancestry from the UK Biobank.^12^ Chronotype was defined by 1 question (“Do you consider yourself to be?”). Participants who considered themselves “definitely a ‘morning’ person” or “more a ‘morning’ than ‘evening’ person” were included as morning persons (n_morning_ = 252,287); participants who considered themselves “definitely an ‘evening’ person” or “more an ‘evening’ than a ‘morning’ person” were included as evening persons as the reference (n_evening_ = 150,908). Participants who responded with “do not know” or “prefer not to answer” were excluded.

#### Insomnia

Insomnia refers to the difficulty in falling asleep or maintaining sleep.^13^ We obtained the genetic associations of insomnia from the GWAS among 237,627 individuals of European ancestry from the UK Biobank.^13^ Insomnia was assessed by self-reports with the question, “do you have trouble falling asleep at night, or do you wake up in the middle of the night?” Individuals who reported “usually” were considered to have frequent insomnia (n_case_ = 129,270), and those who reported “never/rarely” were considered controls (n_control_ = 237,627).

#### Self-reported sleep duration

Self-reported sleep duration refers to the number of hours of sleep an individual get in every 24 hours (including naps).^14^ Genetic associations of self-reported sleep duration were obtained from a GWAS among 446,118 individuals of UK Biobank.^14^ Participants responded to the question “about how many hours sleep do you get in every 24 hours? (please include nap)” with hour increments. Extreme responses of <3 hours or >18 hours were not included in the GWAS analysis. Self-reported sleep duration was used as a continuous variable. Two binary variables were derived from self-reported sleep duration, i.e., short sleep and long sleep. Short sleep was defined as a sleep duration <7 hours, while long sleep was defined as a sleep duration ≥9 hours. Similar cutoffs were used in previous prospective studies on the effect of sleep duration.^15^ The GWAS on short sleep compared the short sleep group (n_ShortSleep_ = 106,192) to the reference group (≥7 and <9 hours of sleep, n_reference_ = 305,742). Accordingly, the GWAS on long sleep compared the long sleep group (n_LongSleep_ = 34,184) to the same reference group as in the GWAS on short sleep.

#### Accelerometer-based sleep-related phenotypes

A triaxial accelerometer device (Axivity AX3, Axivity, Newcastle Upon Tyne, UK) was used continuously for up to 7 days by 103,711 UK Biobank participants.^16^ After the removal of individuals with low measurement quality or poor wear time (<72 hours), data were available for ≈85,000 UK Biobank participants. Genetic associations of accelerometer-based sleep-related phenotypes were obtained from a GWAS among 85,670 individuals of the UK Biobank.^16^ We focused on 4 accelerometer-based sleep-related phenotypes in this study: number of sleep episodes, sleep duration, L5 timing, and sleep efficiency. Individual activity levels based on wrist-worn accelerometer were used to distinguish movement from nonmovement and to estimate the sleep period time (SPT) window. During the SPT window, a period of at least 5 minutes with a change <5° on the z-axis (the dorsal-ventral direction in the anatomic position) was considered 1 sleep episode. A number of sleep episodes <5 or >30 was excluded. Duration of each sleep episode was added up to determine the total sleep duration. Sleep durations <3 hours or >12 hours were excluded. The L5 timing was defined as the 5-hour period with the minimum average acceleration starting from the previous midnight, which indicates the timing when the individuals were asleep. The midpoint of the L5 was used in the GWAS analysis. Sleep efficiency was calculated as sleep duration divided by the duration of the SPT window.

#### Major depressive disorder

Genetic associations with MDD were obtained from the publicly available GWAS among individuals of European ancestry (n_case_ = 9,240 and n_control_ = 9,519) contributed from the Psychiatric Genomics Consortium database (supplementary table e-2, available from Dryad, doi.org/10.5061/dryad.ffbg79cqt).^17^ Cases of MDD were defined as clinically diagnosed MDD according to the DSM-IV.

#### Alzheimer disease

Genetic associations with AD were obtained from the meta-analysis of GWAS on individuals of European ancestry (n_case_ = 21,982 and n_control_ = 41,944) contributed by the International Genomics of Alzheimer's Project (IGAP) (supplementary table e-3, available from Dryad, doi.org/10.5061/dryad.ffbg79cqt).^18^ Details of the IGAP were published elsewhere.^18^ In brief, the meta-analysis comprises 4 GWAS, namely the Alzheimer's Disease Genetics Consortium, the Cohort for Heart and Ageing Research in Genomic Epidemiology consortium, the European Alzheimer's Disease Initiative consortium, and the Genetic and Environmental Risk in Alzheimer's Disease consortium. Either postmortem autopsy or clinical examination was used to evaluate the participants for AD in the individual studies.^18^ Across all cohorts included in the meta-analysis, the mean age at onset of AD in cases ranged from 71.1 to 82.6 years, and the mean age at examination of controls ranged from 51.0 to 78.9 years.^18^

We performed an additional analyses using a larger meta-analysis of AD/AD-by-proxy in individuals of European ancestry (n_case_ = 71,880 and n_control_ = 383,378) (supplementary table e-3, available from Dryad, doi.org/10.5061/dryad.ffbg79cqt).^19^ The meta-analysis comprises GWAS on cases with AD and GWAS on AD-by-proxy cases. GWAS on cases with AD were contributed by the IGAP, Alzheimer work group initiative of the Psychiatric Genomic Consortium, and Alzheimer's Disease Sequencing Project (n_case_ = 24,087 and n_control_ = 55,058). Cases of AD were diagnosed by physician examination or autopsy confirmation. GWAS of AD-by-proxy cases was conducted with UK Biobank data. AD-by-proxy status was based on self-reported parents' diagnoses of AD. A total of 47,793 participants had 1 or both parents affected. AD-by-proxy cases with 2 affected parents were up-weighted. Unaffected parents were weighted by the age or age at death to account for late-onset AD. The author reported a genetic correlation of 0.81 between AD status and AD-by-proxy status.^19^ We used the publicly available summary statistics for the meta-analysis of GWAS on AD and AD-by-proxy cases.

### Statistical analysis

To assess the causal relationship between sleep-related phenotypes, MDD, and AD, we performed bidirectional MR analysis for each pair of traits in this triad. For sleep-related phenotypes and AD, we chose the genetic instruments using genome-wide significant threshold (*p* < 5 × 10^−8^). For MDD, we used an arbitrary threshold of 1 × 10^−4^. We removed correlated single nucleotide polymorphisms (SNPs) (*r*^2^ > 0.1) by keeping the SNP with the smallest *p* value for the association with the exposure. For all exposures, we filtered the instruments for *F* statistics >10 to mitigate potential effects of weak instrument bias using the genotype data.^17^ SNPs were aligned on the basis of their presumed effect allele.

We estimated SNP-specific associations using the Wald ratio (the ratio of the genetic association with the outcome to the genetic association with the exposure).^20^ We combined the SNP-specific associations for MR using inverse variance weighting (IVW).^21^ We also performed sensitivity analyses using weighted median and MR-Egger regression methods to assess the validity of instruments and to investigate influences of potential pleiotropic effects.^21–23^ The MR–pleiotropy residual sum and outlier method was used to identify potential outlier SNPs, which were excluded in the sensitivity analysis.^24^ We accounted for multiple comparisons of 26 groups of associations using a Bonferroni correction (*p* value threshold of 0.05/26 = 0.002).

All statistical analyses were performed with the TwoSampleMR package^25^ in R 3.5.0 (R Foundation for Statistical Computing, Vienna, Austria).

### Data availability

All data used for the analyses are publicly available. Supplementary data are available from Dryad (doi.org/10.5061/dryad.ffbg79cqt).

## Results

In this study, we investigated the causal relationships between sleep-related phenotypes, MDD, and AD by conducting bidirectional MR analysis between each pair of traits. The numbers of instrumental variables (i.e., SNPs associated with exposure) varied for sleep-related phenotypes, MDD, and for AD (supplementary table e-4, doi.org/10.5061/dryad.ffbg79cqt). The full results are available in supplementary table e-5 and supplementary figures e-1 through e-38.

### Sleep and MDD

We did not find causal relationships between sleep-related phenotypes and MDD in either direction (supplementary figures e-1–e-18, doi.org/10.5061/dryad.ffbg79cqt).

### MDD and AD

The MR estimates of the causal relationships between MDD and AD were positive in both directions using MR with IVW, but neither was statistically significant (supplementary figures e-19 and e-20, doi.org/10.5061/dryad.ffbg79cqt).

### Sleep and AD

**Figure 1:**
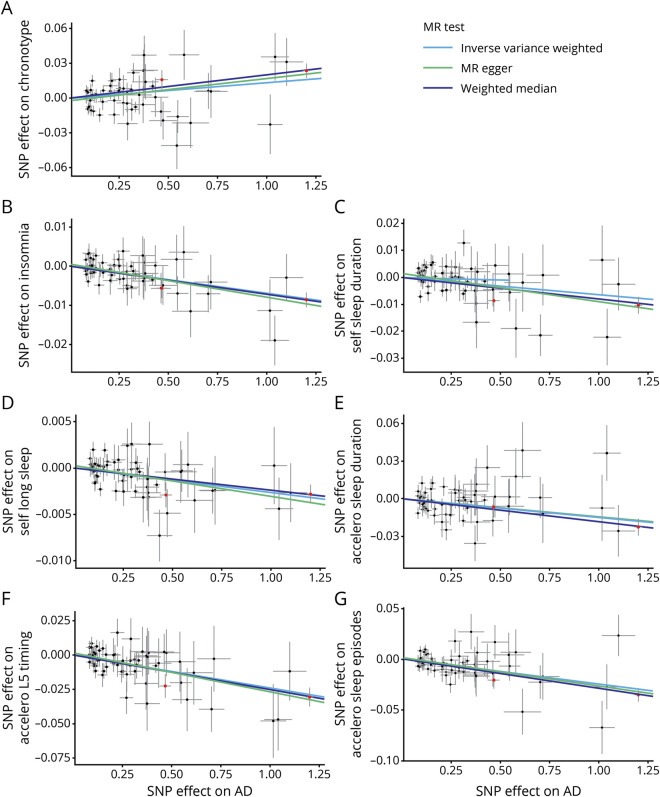
Causal relationships between AD and sleep-related phenotypes in scatterplots (findings adjusted for multiple comparisons) (A) Alzheimer disease (AD) → chronotype; (B) AD → insomnia; (C) AD → self-reported sleep duration; (D) AD → self-reported long sleep; (E) AD → accelerometer-based sleep duration; (F) AD → least active 5 hours (L5) timing; (G) AD → number of sleep episodes (*APOE* single nucleotide polymorphisms [SNPs] [rs7412 and rs429358] are labeled in red). MR = Mendelian randomization.

Genetically higher risks of AD were associated with being a morning person, a lower risk of insomnia, a shorter self-reported and accelerometer-based sleep duration, being less likely to report long sleep, an earlier L5 timing, and a smaller number of sleep episodes after adjustment for multiple comparisons (table 1 and figure 1 and supplementary figures e-21–e-29, available from Dryad, doi.org/10.5061/dryad.ffbg79cqt). MR findings using IVW, weighted median, and MR-Egger were consistent. Results based on the GWAS of AD/AD-by-proxy cases also were similar except that accelerometer-based sleep duration was the only significant sleep phenotype that appeared to be potentially caused by AD when IVW was used after adjustment for multiple comparisons. None of the MR analyses supported a causal effect of sleep-related phenotypes on AD risk (supplementary figures e-30–e-38, available from Dryad).

**Table 1:**
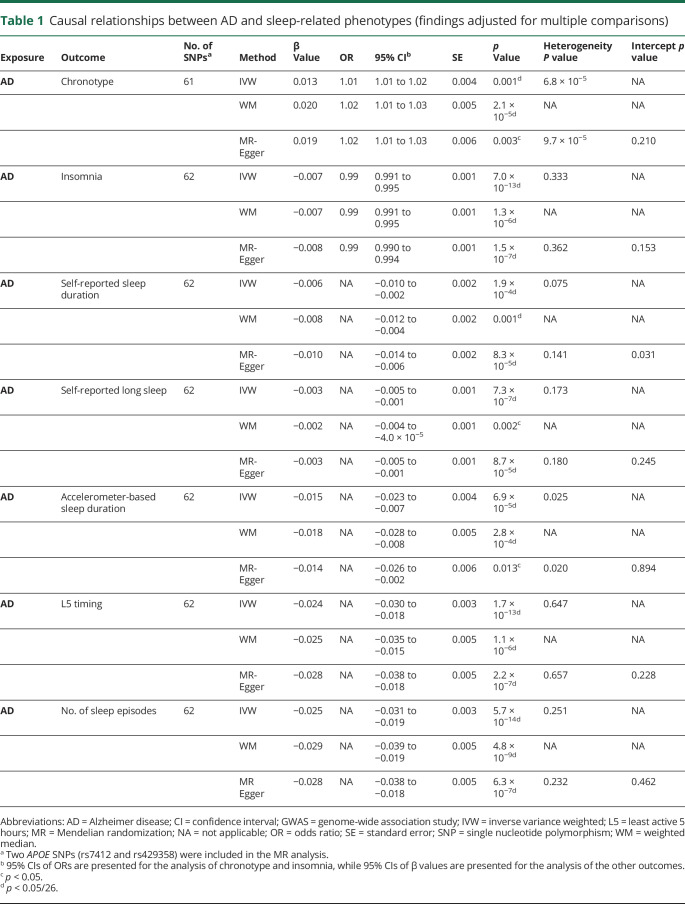
Causal relationships between AD and sleep-related phenotypes (findings adjusted for multiple comparisons)

Because of the larger independent contribution of *APOE* ε4, a gene with pleiotropic effects influencing multiple disease processes that might contribute to AD risk,^26^ we performed a sensitivity analysis excluding *APOE* SNPs (rs7412 and rs429358). Without *APOE* ε4, the causal associations of AD with self-reported and accelerometer-based sleep duration were not significant (supplementary figures e-21–e-29, available from Dryad, doi.org/10.5061/dryad.ffbg79cqt). However, all other causal associations of AD on sleep phenotypes explored here were supported.

## Discussion

A systematic review (supplementary table e-6, available from Dryad, doi.org/10.5061/dryad.ffbg79cqt) found an association between self-reported low sleep quality and depression or depressive symptoms among college students.^27^ Among older adults, the prevalence of coexisting sleep disturbances (defined as insomnia, poor sleep quality, and complaints of insomnia) and depressive symptoms was 10.6%.^28^ In prospective studies among older adults, a bidirectional relationship between sleep disturbances and depression also was reported (supplementary table e-6, available from Dryad).^28^ However, in our study, we did not find evidence for a causal relationship between sleep-related phenotypes and MDD. Apparent inconsistences between our finding and previous observations may be due in part to the different definitions of sleep-related phenotypes. Previous studies considered multiple sleep-related phenotypes together as a global concept of sleep disturbances, while we investigated a number of the commonly characterized sleep-related phenotypes separately. Our concern was that, given the complexity of sleep, the different sleep-related phenotypes provide measures of different elements of sleep behavior; none of the individual phenotypes alone nor their aggregation into a global measure has been validated as a surrogate for the full clinical concept of sleep. The different sleep-related phenotypes also may have individually distinct impacts on human health. Moreover, considered as individual measures, they have a precision that may increase analysis sensitivity.

The comorbidity of depression and AD has been reported widely.^4,5^ Previous systematic reviews (supplementary table e-7, available from Dryad, doi.org/10.5061/dryad.ffbg79cqt) consistently suggested simple associations of depression with both all-cause dementia and AD.^29,30^ In addition, only a trajectory of increasing depressive symptoms was associated with higher risk of dementia.^31^ These findings suggest that depressive symptoms may be a prodrome of AD reflecting preclinical disease progression.^32^ Evidence for associations of MDD and AD at the clinical and molecular levels also has been reported.^9^ Reports have argued that oxidative stress leading to neuronal dysfunction could contribute to the development of both MDD and AD.^9^ The upregulation of brain nuclear factor-kβ and inflammatory cytokine production found in both MDD and AD potentially decreases neuronal proliferation in both diseases.^9^ However, we did not find epidemiologic evidence supporting a causal relationship between MDD and AD. As appropriate genetic instruments become available for late-onset depression, future work should revisit its causal role in AD^33^ because late-onset depression is related more proximately in time to clinical symptoms of AD.

Our study found that a genetically higher risk of AD is associated with several sleep-related phenotypes. Specifically, we found that those who are genetically at higher risk of AD are likely to have lower risk of insomnia and earlier L5 timing. Both the lower risk of insomnia and earlier L5 timing suggest that the individuals were less active in the first half of the night. These results from the study of people at high genetic risk of AD are not consistent with the observational findings of sleep disturbances in people who express AD clinically; e.g., previous studies (supplementary table e-8, available from Dryad, doi.org/10.5061/dryad.ffbg79cqt) describe sleep disturbances as typical symptoms of AD and sleep disordered breathing, excessive daytime sleepiness, and insomnia as frequent sleep disorders in patients with dementia or mild cognitive impairment.^34,35^ Thus, disruption of the sleep-wake cycle—rather than insomnia—is prevalent in patients with AD.^36^ Sleep disturbances also can precede clinical expression of AD.^34^ Both short sleep duration and long sleep duration are associated with a higher risk of a broad range of cognitive disorders, including mild cognitive impairment, dementia, AD, and cognitive decline, suggesting a nonlinear relationship.^37–39^ In our study, we found causal associations of genetically higher risk of AD with shorter self-reported sleep duration, as did the earlier observational studies. While the evidence is not strong, on the basis of these data and our observations, we hypothesize that preclinical stages of AD and more severe, clinically expressed AD may have different effects on sleep phenotypes. These relationships highlight the potential importance of sleep management to improve the quality of life in patients with all stages of AD.

*APOE* is a particularly important genetic contributor to AD risk, alone accounting for ≈13% of the phenotypic variance of AD.^40^ While this may appear to make *APOE* SNPs strong instruments for assessing risk of AD with MR,^10^ because *APOE* is associated with multiple disorders besides AD,^26^ the use of *APOE* SNPs as instruments in an MR analysis for AD violates exclusion restriction assumptions for MR (that genetic instruments affect only the outcome of interest through the risk factor).^10^ While the pleiotropy test from MR-Egger regression suggested little pleiotropy (table 1), we performed an additional sensitivity analysis excluding the *APOE* SNPs. All causal associations of higher risk of AD with sleep patterns were sustained except for the observation of an association with shorter sleep duration. A possible interpretation of these results is that the MR estimate with inclusion of *APOE* may be influenced both by its impact on the genetic risk of AD and through its associations with other disorders.^41^

In this study, we explored the bidirectional relationship in the triad of sleep-related phenotypes, MDD, and AD. The application of MR circumvents the conundrum of unmeasured confounding and reverse causation in observational studies and thus can provide some insights into causal relationships. We have examined the assumptions of MR carefully and selected appropriate data sources for each association under investigation. For example, all SNPs used as instruments have an *F* statistic >10, indicating that weak instrument bias is unlikely. We also performed sensitivity analyses to explore the potential impact of pleiotropic effects of the SNPs used as instruments.

Our study has notable strengths. Specifically, we used data from the largest GWAS available for each trait or disorder under investigation, and we explored a wide range of sleep-related phenotypes, including those objectively estimated with an accelerometer. The objective estimates of sleep may be more precise and are less prone to bias and misclassification than self-reports.^42^ Nevertheless, there are several potential limitations of our study. (1) Overall, we investigated different exposure traits and used different sets of predictive SNPs, but the power of our analyses with the different instruments varies. (2) MR analysis assumes a lifetime exposure to the risk factor and is likely to overestimate the effect of clinical intervention on the outcome,^43,44^ so it cannot be assumed to suggest that an intervention to modify an associated factor will bring clinical benefits (although characterizing any causal relationships can direct hypothesis generation for discovery of biological mechanisms). (3) The most relevant critical exposure period cannot be distinguished with MR because the data available to us for the GWAS for sleep and incidence of AD were obtained only from middle-aged or older individuals (precluding tests of effects of exposure to abnormal sleep patterns in childhood or adolescence). (4) Biological mechanisms underlying different forms of dementia resembling AD may differ,^45^ and only ≈19% of the AD cases used for the GWAS used were autopsy confirmed (moreover, AD subtypes were not taken into account).^18^ (5) *APOE* SNPs may have both direct and indirect (e.g., through their influence on risks of other diseases) effects on risk of AD. (6) Sleep-related phenotypes were either self-reported or estimated with accelerometers rather than measured directly with polysomnography. (7) We used data from individuals of mainly European ancestry, although sleep habits may vary between cultural and ethnic groups. (8) Finally, null findings in our study could reflect lack of power; however, the 2-sample MR design increases statistical power by using multiple data sources,^10^ and our estimates of statistical power (assuming the observed effect and confidence interval in this study represent the true effect sizes)^46^ (supplementary table e-5, available from Dryad, doi.org/10.5061/dryad.ffbg79cqt) suggested that the lack of an effect of long sleep on AD is unlikely to be due to lack of power.

In this study, we found evidence supporting a potential causal influence of AD on sleep disturbances. However, we did not find evidence supporting a causal role of disturbed sleep patterns on AD, suggesting that observed associations between sleep disorders and AD may be due to reverse causation. We did not find evidence for significant causal relationships between MDD and AD. As a first step toward better understanding the basis for observed associations between depression and AD, future work could explore the genetic heterogeneity of depression syndromes to test for causal relationships between potentially etiologically distinct subtypes of depression (e.g., late-onset depression^33^) and AD.

## Glossary

AD: Alzheimer disease
DSM-IV: *Diagnostic and Statistical Manual of Mental Disorders, 4th edition*
GWAS: genome-wide association study
IGAP: International Genomics of Alzheimer's Project
IVW: inverse variance weighting
L5: least active 5 hours
MDD: major depressive disorder
MR: Mendelian randomization
SNP: single nucleotide polymorphism
SPT: sleep period time
